# Use of Artificial Intelligence‐Based Platforms to Support Searches in Evidence Synthesis Studies: A Study of the EAACI Methodology Committee

**DOI:** 10.1002/clt2.70202

**Published:** 2026-09-11

**Authors:** Bernardo Sousa‐Pinto, Antonio Bognanni, Paweł Jemioło, Rafael José Vieira, Thinh H. Nguyen, Zachary Munn, Timothy Hugh Barker, Lemma N. Bulto, Danilo di Bona, Jean Bousquet, Mário Dinis‐Ribeiro, João A. Fonseca, Mariette Awad, Manuel Marques‐Cruz, Holger J. Schünemann

**Affiliations:** ^1^ MEDCIDS – Department of Community Medicine, Information and Health Decision Sciences Faculty of Medicine University of Porto Porto Portugal; ^2^ CINTESIS@RISE – Health Research Network Faculty of Medicine University of Porto Porto Portugal; ^3^ Clinical Epidemiology and Research Center (CERC) Department of Biomedical Sciences Humanitas University Milan Italy; ^4^ Personalized Medicine, Asthma and Allergy IRCCS Humanitas Research Hospital Milan Italy; ^5^ AGH University of Krakow Krakow Poland; ^6^ Chair of Epidemiology and Preventive Medicine Faculty of Medicine Jagiellonian University Medical College Krakow Poland; ^7^ Division of Immunology Boston Children's Hospital Harvard Medical School Boston Massachusetts USA; ^8^ Health Evidence Synthesis, Recommendations and Impact (HESRI) School of Public Health University of Adelaide Adelaide Australia; ^9^ Department of Medical and Surgical Science University of Foggia Foggia Italy; ^10^ Institute of Allergology Charité – Universitätsmedizin Berlin Corporate Member of Freie Universität Berlin and Humboldt‐Universität Zu Berlin Berlin Germany; ^11^ Fraunhofer Institute for Translational Medicine and Pharmacology ITMP Allergology and Immunology Berlin Germany; ^12^ Precancerous Lesions and Early Cancer Management Group Research Center of IPO Porto (CI‐IPOP)/CI‐IPOP@RISE (Health Research Group) Portuguese Institute of Oncology of Porto (IPO Porto)/Porto Comprehensive Cancer Center (Porto.CCC) Porto Portugal; ^13^ Gastroenterology Department Portuguese Institute of Oncology of Porto Porto Portugal; ^14^ Department of Electrical and Computer Engineering American University of Beirut Beirut Lebanon

**Keywords:** allergic rhinitis, artificial intelligence, evidence search, rapid evidence reviews, systematic reviews

## Abstract

**Objective:**

To demonstrate an evaluation of the use of artificial intelligence (AI) in supporting tasks in evidence synthesis (particularly search for primary studies) in the allergy or respirology fields.

**Methods:**

We queried three AI‐based platforms specialised on identifying scientific publications using several strategies (i.e., prompting strategies and search approaches) in November 2024 to identify primary studies related to health‐related case studies in the allergy or respirology field. We compared how many eligible primary studies we identified using AI‐based platforms versus in a systematic search in multiple electronic bibliographic databases. We also compared meta‐analytical results obtained with the primary studies identified by querying AI‐based platforms versus in the context of a systematic review. Finally, we developed a structured methodological framework and a reporting checklist for using these AI‐based platforms.

**Results:**

In our main case study, a systematic review of randomised controlled trials (RCTs) informing the Allergic Rhinitis and its Impact on Asthma (ARIA) guidelines, a strategy involving searching multiple AI‐based platforms identified 85.7% of all full articles with DOI, but failed to identify unpublished trials registered in trial databases, resulting in an overall identification of 56.3% eligible RCTs. Meta‐analytical estimates were similar when considering only the primary studies identified using AI‐based strategies versus those identified by the systematic review. We observed a lower performance in the case study of systematic reviews of observational studies.

**Conclusions:**

We provide an example on how it is possible to evaluate AI‐based platforms in the support of evidence synthesis, with a focus on the allergy and respirology fields.

## 1 Introduction

Authors of evidence synthesis studies are expected to search for eligible data from a variety of sources [1, 2]. In fact, it is highly recommended that searching in electronic bibliographic databases is complemented with other approaches to identifying relevant evidence. While the impact of these complementary approaches might vary based on the type of research question and evidence synthesis effort, they frequently lead to identifying primary studies that would not have been found by solely searching electronic bibliographic databases [3, 4, 5, 6].

Classically, supplementary search methods include searching trial registers and preprint servers, contacting experts and other information sources about unpublished or ongoing studies, checking references of included primary studies and other relevant reviews, and handsearching journals and grey literature sources [1, 3]. More recently, artificial intelligence (AI)‐based platforms specialised on the identification of scientific publications have opened the possibility for new search approaches to support evidence synthesis. In particular, these platforms can identify and summarise scientific articles relevant to specific research questions. These functions can potentially assist authors of evidence synthesis studies to test their queries, complement their searches or in exceptional rapid review situations, replace the use of other sources. However, the application of these platforms in evidence synthesis studies has not yet been evaluated [7]. Consequently, to understand what role these platforms might play, it is essential to investigate their capacity to identify relevant studies, and develop a methodological framework for their use.

In this study, we aimed to demonstrate an evaluation on the use of specialised AI‐based platforms to support the search for primary studies in evidence synthesis, with case studies in allergy or respirology fields. While this a rapidly evolving field and our evaluation dates from November 2024, we aim that this study provides an example on how we can evaluate the use of AI‐based platforms in evidence synthesis and, based on that, propose structured and reproducible methodological frameworks, and checklists of reporting items. Our main case study is part of a systematic review [8] that informed the Allergic Rhinitis and its Impact on Asthma (ARIA)—European Academy of Allergy and Immunology (EAACI) 2024‐2025 guidelines [9]. We evaluated additional case studies in the allergy and respiratory fields.

## 2 Methods

### 2.1 Study Design

In November 2024, we queried three platforms specialised in using AI to identify scientific articles, namely Elicit, Scispace and Scite. For terminology purposes, a “query” corresponds to the introduction of a search expression in an AI‐based platform (structured through a prompt), while “strategy” refers to the whole set of approaches used to identify studies in AI‐based platforms (henceforth, a “strategy” may involve applying one or multiple queries to one or multiple AI‐based platforms).

As our main case study, we applied different queries to each platform to identify randomised controlled trials (RCTs) investigating mometasone in patients with allergic rhinitis. Then, we evaluated the performance of a strategy involving the search of multiple platforms using different queries (akin to searching multiple electronic bibliographic databases). Specifically, and adopting a relative recall approach [10], we evaluated how many of the RCTs that were originally included in a previously completed systematic review on mometasone for allergic rhinitis, would be identified by that strategy [8]. In addition, we conducted meta‐analyses and assessed the certainty of evidence when considering only the RCTs identified based on that AI‐based search strategy, and compared the obtained results with those described in our systematic review [8]. In order to assess the robustness of our results, we assessed multiple other strategies. Specifically, we evaluated the results obtained in strategies (i) querying each platform individually, (ii) indicating or not the outcome of interest in the search queries, or (iii) considering or not identifying relevant systematic reviews. In addition, we also evaluated other case studies (i.e., search for RCTs on other allergy medications, and search for non‐randomised observational studies on an association between an exposure and an outcome).

### 2.2 Main Case Study

For our main case study, we sought to identify via AI‐based platforms the RCTs on mometasone for allergic rhinitis included in a systematic review conducted by our team, described in detail elsewhere [8], and used to inform the ARIA‐EAACI 2024‐2025 guidelines [9]. In brief, we included parallel RCTs comparing mometasone versus placebo in adult patients with allergic rhinitis who have reported at least one of the following outcomes: (i) total nasal symptom score, (ii) total ocular symptom score, (iii) total symptom score, and (iv) rhinoconjunctivitis quality‐of‐life questionnaire. Overall, in that systematic review, we included 32 RCTs on mometasone, of which (i) 21 were full papers with a digital object identifier (DOI), (ii) 1 was a full paper without a DOI, (iii) 1 was a conference abstract, and (iv) 9 were unpublished clinical trial registrations with available results.

#### 2.2.1 Information Sources: AI‐Based Platforms

We queried three platforms that use AI to identify scientific articles: Elicit (elicit.com), Scispace (typeset.io) and Scite (scite.ai). These platforms identify and summarise retrieved scientific articles when provided with a query structured as a question. We selected these specific platforms in order to ensure diversity in terms of focus, algorithms and proprietary company. We used the freely available features of these platforms (except for Scite, which does not have a free version). Supporting Information S1: Table S1 provides a description of these platforms by the time they were queried, displaying their similarities and differences.

#### 2.2.2 Article Search and Retrieval

For each case study, we queried each AI‐based platform with four different prompts in November 2024. In brief, the four queries/prompts differed in the type of studies searched and in whether information on the relevant outcomes was requested. The four prompts displayed the following structure (based on the Population‐Intervention‐Comparison‐Outcome [PICO] format):

- “Indicate parallel randomised controlled trials comparing mometasone versus placebo in adult patients with allergic rhinitis”;
- “Indicate parallel randomised controlled trials comparing mometasone versus placebo in adult patients with allergic rhinitis on nasal symptoms, ocular symptoms or quality of life”;
- “Indicate systematic reviews comparing mometasone versus placebo in adult patients with allergic rhinitis”;
- “Indicate systematic reviews comparing mometasone versus placebo in adult patients with allergic rhinitis on nasal symptoms, ocular symptoms or quality of life”.

We applied different article retrieval approaches according to whether platforms were queried to identify RCTs or systematic reviews, particularly considering that the objective of searching for systematic reviews was to identify additional sources of potentially eligible records. When we queried platforms to identify RCTs, we retrieved the first 50 results displayed by each platform for each query. When we queried for systematic reviews, one researcher (i) manually screened (by title and—if needed—abstract) the first 50 results displayed by each platform for each query, and (ii) retrieved those corresponding to systematic reviews answering to the respective research question. We then identified all unique references of the retrieved systematic reviews by using citationchaser [11] (considering the use of this automated tool, the list of references that we gathered was not restricted to the primary studies included in the retrieved systematic reviews but rather contained all their references). This procedure was done because not all obtained results corresponded to relevant systematic reviews and retrieving the references of all 50 results would result in a potentially large number of irrelevant records. Of note, even though it appeared on platform results, we did not retrieve the systematic review conducted by our team [8], as its results were used as “ground truth” to assess the performance of our search strategies. On another note, the cap of assessing the 50 results per query was a methodological predefinition defined prior data retrieval, with the goals of ensuring reproducibility and that the same stopping criterion would be employed by all researchers and in all searched platforms.

#### 2.2.3 Evaluation of Strategies for Identification of Articles

After article retrieval, we identified the number of unique records considering all applied queries across all searched platforms (main strategy). We assessed, among that set of unique records, how many RCTs included in our systematic review were identified by the AI‐based platforms. Subsequently, we conducted a quantitative evidence synthesis via meta‐analysis and appraisal of the certainty of evidence across the included RCTs, and compared the outputs with the meta‐analytical results and appraisal judgements from the original review. We performed meta‐analysis by considering the same effect size measures, models and methods as in the original review (i.e., random‐effects meta‐analysis of mean differences with τ^2^ estimated using the restricted maximum likelihood approach). We used the Grading of Recommendations Assessment, Development and Evaluation (GRADE) approach for appraisal of the certainty of evidence (https://book.gradepro.org/).

In order to assess the impact of (i) querying multiple AI‐based platforms (i.e., considering each platform individually instead of collectively), (ii) indicating the outcomes of interest in the queries, and (iii) searching for systematic reviews in addition to RCTs, we tested the results that would be obtained if other strategies have been applied.

Finally, 1 month after having queried AI‐based platforms, we queried them again, repeating all described methodological procedures. This allowed us to assess the short‐term consistency of obtained results if searching the same platforms with the same queries more than once.

### 2.3 Additional Case Studies

To assess the robustness of our results, we applied the process above to three additional case studies (i.e., to other research questions). Two of these case studies (additional case studies 1 and 2) implied identifying RCTs on anti‐allergic medications of other classes, namely olopatadine and olopatadine‐mometasone [8]. Differently, additional case study 3 concerned an exposure: we queried AI‐based platforms to identify non‐randomised observational studies assessing diabetes as a risk factor for tuberculosis, comparing our results with those of the systematic review by Franco et al. [12]. Of note, all primary studies of additional case studies 1–2 and most of those of additional case study 3 corresponded to full papers with DOI.

As for the main case study, AI‐based platforms were queried in November 2024 for additional case studies 1–2. For additional case study 3, AI‐based platforms were queried in October 2025 by a research team external to that which developed the methodological framework for searching AI‐based platforms. The prompts used to query AI‐based platforms displayed the same structure as those used for our main case study.

### 2.4 Development and Testing of the Methodological Framework and of the Reporting Checklist

Based on our experience in this study, we developed (i) a methodological framework for the use of AI‐based platforms in evidence search, and (ii) a checklist with reporting items for authors to report searches based on those platforms. Four authors jointly drafted a first version of the framework and of the checklist. Subsequently, we created an online survey so that, for each item, each author could vote on whether he/she would (i) agree with it, (ii) suggest some rewording, or (iii) disagree with it. In addition, authors were able to suggest new items. Finally, we sent a revised version of the framework and of the checklist to all co‐authors, so that consensus could be reached.

The framework was piloted and tested by a research team that was not involved in its development. That research team applied the framework to additional case study 3, providing further feedback.

## 3 Results

### 3.1 Main Case Study

#### 3.1.1 Main Strategy

**FIGURE 1:**
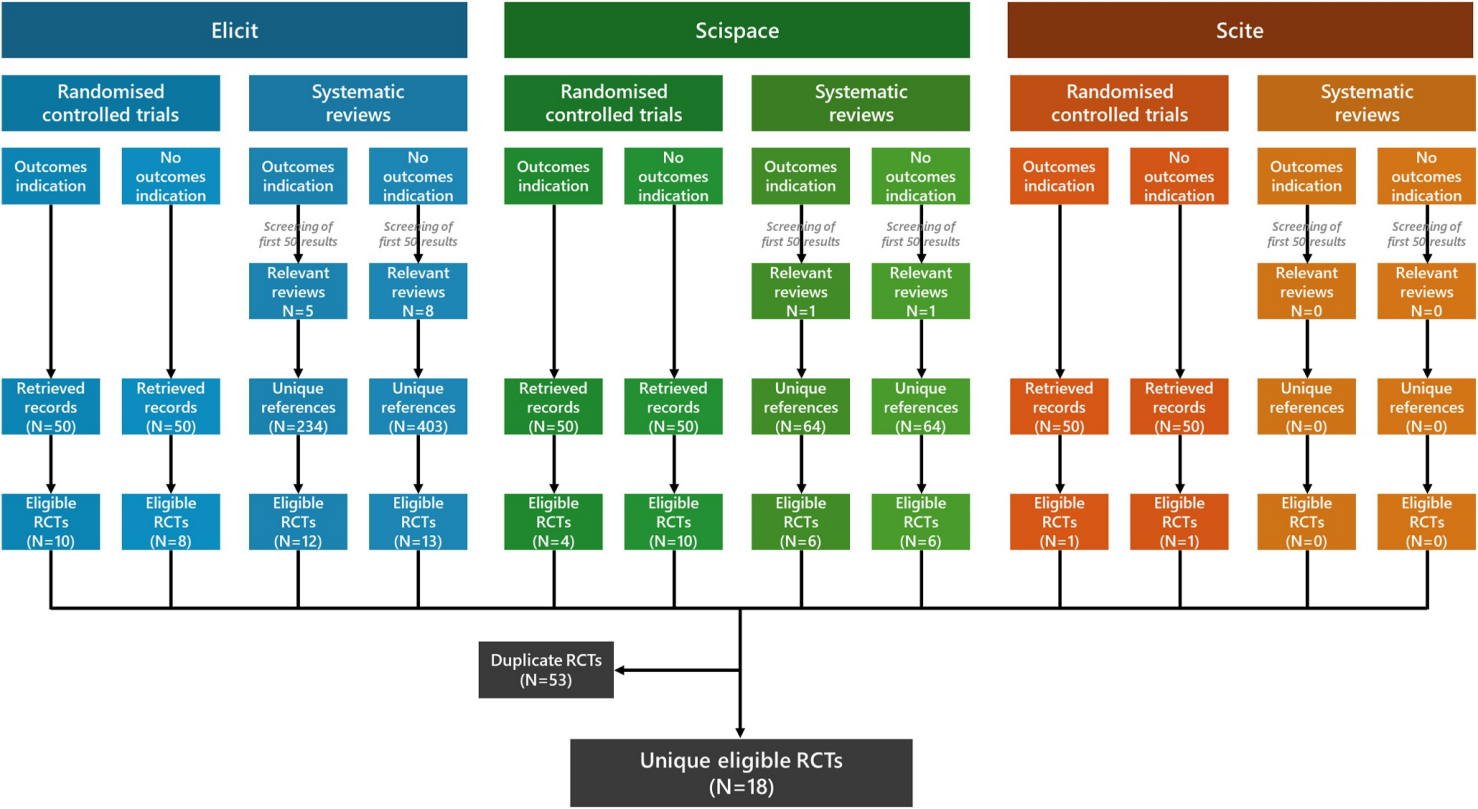
Flow diagram illustrating the process of identification of eligible randomised controlled trials (RCTs) according to the main strategy.

With our main AI‐based strategy, we were able to identify 18 eligible RCTs comparing mometasone versus placebo in adults with allergic rhinitis (out of a total of 592 unique retrieved records) (Figure 1). This corresponds to 56.3% of all RCTs on mometasone included in our systematic review and to 85.7% of all full papers with a DOI (Table 1). None of the eligible RCTs that had not been published as a full paper with a DOI was identified by our main AI‐based strategy.

**TABLE 1:** Number of primary studies found with the main strategy and with alternative strategies for searching AI‐based platforms.

Platform	Study designs being queried	Queries with outcome indication	*N* unique records	*N* eligible primary studies found	% of all primary studies (*N* = 32)	% of full papers with DOI (*N* = 21)
Main search strategy						
All	RCTs + systematic reviews	Combination^a^	592	18	56.3	85.7
Additional search strategies						
All	RCTs + systematic reviews	No	466	17	53.1	81.0
All	RCTs + systematic reviews	Yes	332	17	53.1	81.0
All	RCTs	Combination^a^	194	17	53.1	81.0
All	RCTs	No	126	14	43.8	66.7
All	RCTs	Yes	126	12	37.5	57.1
Elicit	RCTs + systematic reviews	Combination^a^	477	18	56.3	85.7
Elicit	RCTs + systematic reviews	No	398	17	53.1	81.0
Elicit	RCTs + systematic reviews	Yes	260	17	53.1	81.0
Elicit	RCTs	Combination^a^	65	11	34.4	52.4
Elicit	RCTs	No	50	8	25.0	38.1
Elicit	RCTs	Yes	50	10	31.3	47.6
Scispace	RCTs + systematic reviews	Combination^a^	138	13	40.6	61.9
Scispace	RCTs + systematic reviews	No	104	12	37.5	57.1
Scispace	RCTs + systematic reviews	Yes	111	10	31.3	47.6
Scispace	RCTs	Combination^a^	80	11	34.4	52.4
Scispace	RCTs	No	50	10	31.3	47.6
Scispace	RCTs	Yes	50	4	12.5	19.0
Scite	RCTs + systematic reviews	Combination^a^	85	1	3.1	4.8
Scite	RCTs + systematic reviews	No	50	1	3.1	4.8
Scite	RCTs + systematic reviews	Yes	50	1	3.1	4.8
Scite	RCTs	Combination^a^	85	1	3.1	4.8
Scite	RCTs	No	50	1	3.1	4.8
Scite	RCTs	Yes	50	1	3.1	4.8

Abbreviations: DOI = digital object identifier; RCT = randomised controlled trials.

^a^ Results obtained when considering records obtained both with queries indicating and not indicating the outcomes of interest.

Table 2 displays the meta‐analytical results obtained with the RCTs identified by our main strategy. Meta‐analytical point estimates were similar to those obtained in our systematic review, but they were associated with higher imprecision. For one of the outcomes, we also observed substantially higher heterogeneity (*I* ^2^ = 71% vs. 49%). No major differences were observed in terms of certainty of evidence (Supporting Information S1: Table S2). That is, when not considering the possibility of publication bias, the certainty of evidence for all outcomes would be the same as in our original systematic review [8]. The only exception would be nasal symptoms in seasonal allergic rhinitis, which would be associated with “High” certainty of evidence following our AI‐based strategy versus “Moderate (downgraded due to inconsistency)” in the original systematic review.

**TABLE 2:** Meta‐analytical results (i) of the systematic review, and (ii) obtained with the main search strategy.

Outcome	Meta‐analytical results—systematic review [8]: MD (95% CI) [*I* ^2^] [*N* studies]	Meta‐analytical results—main search strategy: MD (95% CI) [*I* ^2^] [*N* studies]
TNSS (SAR)	−0.95 (−1.12; −0.79) [47%] [17]	−0.98 (−1.22; −0.75) [43%] [8]
TOSS (SAR)	−0.30 (−0.48; −0.12) [0%] [3]	−0.36 (−0.58; −0.14) [0%] [2]
RQLQ (SAR)	−0.38 (−0.55; −0.20) [49%] [6]	−0.46 (−0.77; −0.14) [71%] [3]
TNSS (PAR)	−1.70 (−3.70; 0.30) [59%] [3]	−2.40 (−4.80; 0) [−] [1]

Abbreviations: CI = confidence interval; MD = mean difference; PAR = perennial allergic rhinitis; RQLQ = rhinoconjunctivitis quality of life questionnaire; SAR = seasonal allergic rhinitis; TNSS = Total Nasal Symptom Score; TOSS = Total Ocular Symptom Score.

#### 3.1.2 Additional Strategies

Querying multiple platforms for RCTs only (i.e., without considering references of systematic reviews) resulted in the identification of at most 17 RCTs (53.1% of all eligible trials; 81.0% of all papers with a DOI) (Table 1). For some platforms, querying one platform only led to similar results to querying multiple platforms when searching for both RCTs and systematic reviews, but not when searching for RCTs only. In this last scenario, we were able to identify at most 11 RCTs (Elicit and Scispace). Being explicit about the outcomes of interest in the queries had variable impact on the obtained results: in Elicit, it was associated with identification of a higher number of RCTs, while in Scispace the opposite effect was observed. The meta‐analytical results that would have been obtained with the different strategies are displayed in Supporting Information S1: Table S3.

#### 3.1.3 Consistency of Results When Querying AI‐Based Platforms at Different Time Points

One month after having queried AI‐based platforms (December, 2024), we queried them again. Applying our main strategy, this resulted in the identification of one additional RCT compared to the previously identified 18 records (Supporting Information S1: Table S4). The inclusion of this additional trial would result in minimal changes to meta‐analytical measures (Supporting Information S1: Table S5).

For most of the additional strategies, querying AI‐based platforms in different time points resulted either (i) in the identification of the exact same eligible RCTs or (ii) in a difference of one eligible RCTs identified (Supporting Information S1: Table S4).

#### 3.1.4 Implementation Considerations

When querying for RCTs, we needed on average 7 minutes to retrieve the first 50 records of each query in each platform. On the other hand, when querying for systematic reviews, we needed on average 16 minutes until obtaining the references of relevant reviews identified by applying each query in each platform. This corresponds to (i) 13 minutes for screening the first 50 records for relevant systematic reviews, and (ii) 3 minutes for identification of unique references in citationchaser and compilation in a Microsoft Excel dataset for purposes of this study. Of note, this does not reflect the time required to learn how to use the AI‐based platforms or citationchaser.

When querying AI‐based platforms, results were presented sorted by relevance. For some queries on RCTs (applied in individual AI‐based platforms), additional eligible results were still being identified by the time that 50 records had been retrieved (Supporting Information S1: Figure S1). While we have predefined the number of results to evaluate following each query, an alternative approach would involve retrieving records until ceasing to identify relevant RCTs.

### 3.2 Additional Case Studies

With our main AI‐based strategy, we were able to identify all RCTs included in additional case studies 1 and 2 (Supporting Information S1: Table S6).

A different result was observed in additional case study 3 (Supporting Information S1: Table S7), as we were able to identify only 17 of the 48 observational primary studies included in the respective systematic review. The identified records tended to be older and more explicit about evaluating the association between diabetes in tuberculosis in the title or abstract compared to non‐identified records (Supporting Information S1: Table S8). Using the identified primary studies, we calculated meta‐analytical results for the main analyses, reporting hazard ratios or risk ratios, but not odds ratios (Supporting Information S1: Table S9).

### 3.3 Proposed Methodological Framework and Reporting Checklist

**FIGURE 2:**
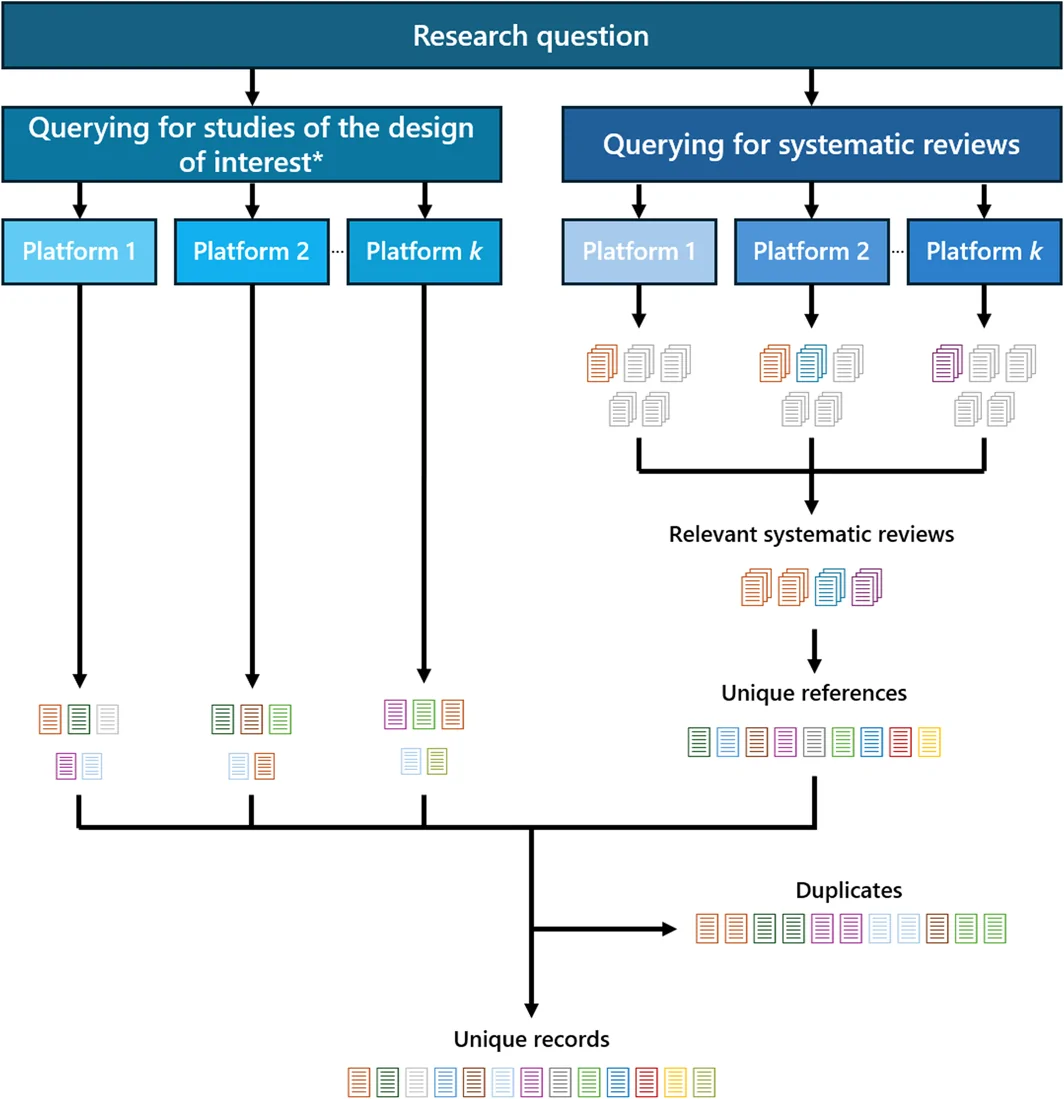
Graphical representation of the proposed methodological framework for the use of artificial intelligence (AI)‐based platforms for searches in evidence synthesis studies *In reviews of RCTs, authors may explicitly mention the study design in their queries.

The proposed methodological framework for the use of AI‐based platforms in searches for evidence synthesis studies is displayed in Box 1, while its graphical representation is available in Figure 2. The checklist with reporting items is available in Box 2, with supplementary Box 1 providing an explanation and example for each item.

**BOX 1 Methodological framework for the use of artificial intelligence (AI)‐based specialised platforms in searches for evidence synthesis studies.**

1

1. Selection of AI platforms:
  1.1. Query AI‐based platforms that have been specifically designed to identify scientific articles when prompted with a research question;
  1.2. Multiple AI‐based platforms should be queried, in order to maximise the sensitivity of the search strategy.
2. Prompting AI‐based platforms:
  2.1. Prompts to AI‐based platforms should be structured based on the question authors aim to address. If the PICO structure is adopted, these prompts should reference, as adequate and possible, the population(s)/problem(s), intervention(s)/exposure(s), comparison(s) and outcome(s) of interest;
  2.2. Authors may opt to query each AI‐based platform with multiple distinct prompts in order to maximise the sensitivity of the search strategy ^a^. In that case, consistency of the results may be evaluated.
3. Consideration of evidence from studies with the design(s) of interest:
  3.1. For systematic reviews of randomised controlled trials, AI‐based platforms should be queried for studies mentioning the study design. Prompts should be structured according to the recommendations displayed in 2.1. and 2.2;
  3.2. Before AI‐based platforms are queried, authors should define whether they will retrieve a predefined number of records or whether they will be retrieving records until ceasing to identify relevant results^b^.
4. Consideration of evidence from published systematic reviews:
  4.1. AI‐based platforms should be queried for systematic reviews that have assessed the question of interest. Prompts should be structured according to the recommendations displayed in 2.1. and 2.2;
  4.2. Results returned by AI‐based platforms should be assessed to make sure that they (i) are systematic reviews, and (ii) address the research question of interest. All eligible systematic reviews should be selected so that the references of these reviews can be retrieved;
  4.3. Before AI‐based platforms are queried, authors should define whether they will screen a predefined number of results or whether they will be screening results until they cease to identify relevant systematic reviews ^b^;
  4.4. Authors should obtain a list of unique references of all retrieved systematic reviews: authors should gather the list of all references of each eligible systematic review and perform deduplication, so that a list of unique references is obtained. Proper software can be used to support that process through automation of reference gathering (without requiring the full text of systematic reviews) and deduplication;
  4.5. Depending on the publication year of reviews and of the time and resources available to the research team, authors may also consider obtaining a list of papers that have cited the retrieved systematic reviews ^c^. Proper software can be used to support that process through automation of reference gathering (without requiring the full text of systematic reviews) and deduplication.
5. Complementary strategies:
  5.1. This search strategy should be complemented by searches on grey literature platforms (e.g., clinical trial registration databases) ^c^. However, this complement depends on the time and resources available to the research team, and on the capacity of the platforms or models used to search grey literature.

^a^ This item concerns interacting with each AI‐based platform or model in different ways (e.g., prompting them with different words) for each question being addressed. This item is not about having different (PICO) questions; ^b^ This definition should take into account factors such as (i) whether it is possible to have an estimate of the expected amount of eligible studies (i.e., whether the question of interest has been subject of intense research; often difficult to guess particularly in emergent new conditions), (ii) available resources, and (iii) the fact that platforms tend to sort their records by apparent relevance using black box models; ^c^ The rationale for this indication and practical information can be found in the Cochrane Handbook for Systematic Reviews of Interventions and the corresponding technical supplement [1].

**BOX 2 Checklist with reporting considerations for authors using artificial intelligence (AI)‐based specialised platforms for evidence search.**

1 Authors should be fully transparent in their use of AI‐based platforms for evidence search. In particular, authors should comply with the following indications (does not exclude the need for complying with more general statements such as the Preferred Reporting Items for Systematic reviews and Meta‐Analyses [PRISMA] and its extension for reporting literature searches [PRISMA‐S]):

1. Identify and justify the rationale and purpose for which AI‐based platforms were used for evidence search (validation, complement or replacement of search in more traditional sources);
2. Specify all AI‐based platforms queried—and, if available, their underlying models—and search dates;
3. Provide all the prompts used to query AI‐based platforms;
4. Indicate whether there was a search for relevant systematic reviews and a subsequent retrieving of their references;
5. Indicate and justify what was the number of records retrieved or screened from querying each platform;
6. Describe the results of the search process, providing the number of obtained records, the number of unique records and the number of relevant eligible studies.

The framework and the reporting checklist are expected to be updated in the future, reflecting (i) future advances on AI and (ii) feedback sent by end‐users.

## 4 Discussion

This study provides a demonstration on how it is possible to evaluate the use of AI to support evidence synthesis and, based on that, to produce frameworks and reporting checklists for responsible use of AI. To perform this study, we considered four case studies in the allergy or respiratory fields. In our main case study, a strategy combining the querying (i) of multiple platforms and (ii) of both primary studies and systematic reviews was able to identify most full papers with DOIs that addressed the underlying research question but failed to identify non‐DOI grey literature. On the other hand, a lower proportion of relevant studies was identified in the case study of a systematic review of non‐randomised observational studies. Importantly, our assessments were conducted in November 2024 and evolutions in the field may have resulted in some of the limitations that we identified in these platforms to have been overcome. In any case, the methods applied in this study can be followed by other retrospective evaluations of AI‐based platforms or workflows.

Based on the assessed case studies, we found that querying multiple AI‐based platforms yields higher search sensitivity than querying a single platform. In fact, searching the different platforms yielded different results, which may reflect differences in their corpora (Supporting Information S1: Table S1) and, most importantly, in their algorithms and search features. This can be particularly relevant considering that a small number of primary studies can often decisively change meta‐analytical results. This impact can be particularly high for questions for which there is a small number of available primary studies: in our case studies, a noteworthy example is that of the total nasal symptom score in perennial allergic rhinitis, which has only been assessed by three RCTs, of which were only able to find one. Finally, we obtained evidence that, when querying AI‐based platforms, searching for systematic reviews and retrieving their references can be an important complement, potentially resulting in an increased amount of identified primary studies. Several of these findings have a parallel with recommended methodological practices for searching electronic bibliographic databases when gathering evidence for systematic reviews [1].

We observed a higher percentage of identified relevant primary studies for systematic reviews of RCTs than of non‐randomised observational studies. The underlying reasons may include the wider diversity of observational studies and the less homogeneous way in which they are described (e.g., often not being explicit about their study design in their title or abstract). This is consistent with what occurs with traditional search approaches, in which searching for observational studies is considered more challenging than searching for RCTs [13]. Future studies may evaluate whether it is possible to optimise the capacity of AI‐based platforms to identify non‐randomised observational studies, whether such capacity varies with the specific research question being addressed, and whether advances in the field have led to an improvement in the capacity to identify this type of study.

The role of AI‐based platforms may vary according to existing time and resource constraints. That is, authors may query AI‐based platforms to test, complement, or—*in extremis*—replace their searches in more traditional sources. Building search queries for bibliographic databases is an iterative process and it is recommended that authors test the queries they have built by assessing whether they are able to return a set of pre‐specified key references [14]. Using AI‐based platforms may allow authors to identify a larger set of such key references (so that they can more adequately test their queries in electronic bibliographic databases). In a different perspective, authors may query AI‐based platforms as an additional method for searching for evidence. That is, querying AI‐based platforms can be used to complement more traditional search methods, such as searching electronic bibliographic databases or clinical trials registries. By contrast, the use of AI‐based platforms to replace searches in other “more traditional” sources should be restricted to rapid reviews of RCTs and in contexts of severe resource constraints. In fact, the gains in efficiency should not only be weighed against the possibility of missing relevant studies but also against the lack of full replicability of these strategies (as they rely on non‐deterministic, black‐box models) and against the uncertainty in the functioning and evolution of the platforms' algorithms. In particular, and considering our results, authors should be particularly aware that AI‐based platforms can fail to identify studies that are only available in grey literature sources such as clinical trial registration databases. This may contribute to dissemination bias, a phenomenon that systematic reviews should try to minimise. Finally, in living systematic reviews of RCTs, regularly querying AI‐based platforms may help authors determine whether a search update is needed. In any case, whatever the purpose for which AI‐based platforms are used, authors should be fully transparent about their choices [15]. Moreover, such choices should be defined at the protocol stage before authors start to query those platforms.

Building on our experience in this study, we developed a methodological framework and, to support its use in a more transparent manner, a checklist of reporting items. The framework we developed is only suitable for individually searching platforms specialised in supporting literature searches and should not be applied to general AI‐based chatbots that present less‐structured results. While advances in the field have opened the possibility to use these platforms differently (e.g., with research agent workflows) such approaches have not been formally tested.

This study has some limitations. Firstly, all our case studies concern the allergy and respiratory field, potentially posing some generalisability concerns to other areas. Even in the allergy field, the assessed cases studies concern respiratory allergy. While the proposed framework and checklist would be applicable to other fields, the performance of AI‐based tools to identify relevant results needs to be explored. In addition, our main case study addressed a research question for which there was abundant grey literature, including articles published in languages other than English, conference abstracts, and results of clinical trials not published in journal articles. We predefined that we would be retrieving the first 50 results displayed by each platform for each query, but in some cases it was possible that additional eligible RCTs would have been found if we would have set a higher limit in the number of results to evaluate (or if we determined that we would be evaluating results until no new eligible RCT would be identified). That is, the 50‐result limit may have led to an underestimation in the percentage of eligible RCTs found by AI‐based platforms. Another limitation concerns the different time points when searches for this study vis‐à‐vis those for the original systematic reviews were made: since publications become more retrievable over time, it is possible that the proposed framework may fail to identify studies that are very recent. Unfortunately, our approach was unable to test that or account for the differences related to when searches were made. As an additional limitation, we did not test whether querying AI‐based platforms resulted in the identification of eligible primary studies that had not been included in the original systematic reviews. Evaluating that retrospectively could make the study even more prone to the limitations of the relative recall method. For example, if the search queries of the reference standard were poorly built (for transparency purposes, the assessment of the search query used in the main case study is available in Supporting Information S1: Table S10), there could be an overestimation of the potential impact of AI‐based platforms in complementing searches through more traditional means. An approach that could circumvent this limitation would consist of a prospective evaluation in which querying AI‐based platforms are used alongside “traditional” methods for evidence search, with the added value of the former being assessed. One other limitation concerns the fact that, despite querying multiple AI‐based platforms, we have not searched all such platforms. In fact, our primary goal was not to compare the different platforms, and in evidence syntheses studies authors also do not search all bibliographic databases.

An important limitation concerns the fact that this evaluation concerns the use of individual AI‐based platforms as of November 2024. The use of AI to support evidence synthesis is a quickly evolving field and other approaches have emerged, such as the possibility of using general‐purpose large language models connected to multiple sources, including but not restricted to the aforementioned platforms. However, an adequate assessment of such workflows would necessarily be a prospective one (i.e., application of these workflows in the context of a systematic review being conducted *de novo*, with comparison of their results with those independently obtained when searching evidence through traditional means), as replicating our retrospective approach with such workflows could lead to optimistic results. i.e., we would not be able to exclude the possibility that the systematic reviews that served as reference standard for each case study would serve as a source for primary studies that these workflows would identify. Such information sources would not be available in real settings where authors are conducting systematic reviews *de novo* and there are no other updated evidence syntheses addressing the same research question.

This study has also several strengths. We have performed a demonstration on how to methodologically evaluate the use of AI to support tasks in evidence syntheses. In our case studies, we evaluated the impact of considering different study designs and, for each study design, we have tested two variants for each. This approach is in line with the one adopted in previous studies assessing the use of AI in evidence‐based practice‐related tasks (e.g., formulation of guideline questions) [16]. Additionally, we not only evaluated the number of eligible primary studies that we were able to identify, but we also assessed the impact of our search strategy on meta‐analytical results. Therefore, we were able to quantify the impact resulting from conducting a search strategy solely based on querying AI‐based platforms. For our main case study, meta‐analytical results and certainty of evidence assessments were similar when considering all studies identified in the original systematic review vis‐à‐vis only those identified based on AI‐based platforms. Therefore, adopting the latter strategy would probably not have a major impact in guideline recommendations in terms of desirable and undesirable effects. However, it should be stated that these are not the only criteria considered when developing a guideline recommendation. For other usually considered criteria—for example, values, resources required, cost‐effectiveness and equity [17, 18], the performance of searches in AI‐based platforms has not been evaluated.

In conclusion, this study demonstrates an evaluation of the use of AI‐based tools or processes in supporting evidence synthesis tasks, and how the results of such evaluations can support the creation of frameworks and checklists supporting a structured and transparent use of AI. Our results—based on approaches to search standalone AI‐based platforms—suggest that querying AI‐based tools can support searching in evidence synthesis studies, at least in the allergy or respirology fields and with potential value when primary studies consist of RCTs. In addition, these tools can be used as a quick quality check step for iterative testing of search queries for electronic bibliographic databases.

## Author Contributions

**Bernardo Sousa‐Pinto:** conceptualization, data curation, formal analysis, writing – original draft, methodology. **Antonio Bognanni:** conceptualization, methodology, writing – original draft. **Paweł Jemiolo:** conceptualization, methodology, writing – original draft. **Rafael José Vieira:** conceptualization, writing – review and editing. **Thinh H. Nguyen:** methodology, writing – review and editing. **Zachary Munn:** data curation, writing – review and editing, formal analysis. **Timothy Hugh Barker:** writing – review and editing, formal analysis, data curation. **Lemma N. Bulto:** data curation, formal analysis, writing – review and editing. **Danilo di Bona:** methodology, writing – review and editing. **Jean Bousquet:** methodology, writing – review and editing. **Mário Dinis‐Ribeiro:** methodology, writing – review and editing. **João A. Fonseca:** methodology, writing – review and editing. **Mariette Awad:** methodology, writing – review and editing. **Manuel Marques‐Cruz:** conceptualization, writing – review and editing. **Holger J. Schunemann:** methodology, writing – review and editing.

## Funding

The authors have nothing to report.

## Ethics Statement

The authors have nothing to report.

## Conflicts of Interest

The authors declare no conflicts of interest.

## Institutional Review Board Statement

The authors have nothing to report.

## Supporting information


Supporting Information S1

## Data Availability

The data that support the findings of this study are available from the corresponding author upon reasonable request.
