# Supplementary material for: Use of Artificial Intelligence‐Based Platforms to Support Searches in Evidence Synthesis Studies: A Study of the EAACI Methodology Committee
Source: Clin Transl Allergy. 2026 Sep 11;16(9):e70202. doi: 10.1002/clt2.70202 (PMC13569465; doi:10.1002/clt2.70202)
Supplement: Supplementary file 1 [file CLT2-16-e70202-s001.docx]

**Supplementary material: Use of Artificial Intelligence-based platforms to support searches in evidence synthesis studies**

**Supplementary Table 1. Characteristics of the queried artificial intelligence (AI)-based platforms.** Information was retrieved from the webpage of each platform (Elicit: elicit.com; Scispace: typeset.io; Scite: scite.ai) as of November 2024.

| **Features** | **Elicit** | **Scispace** | **Scite** |
| --- | --- | --- | --- |
| Main purposes and focus | Identification of relevant papers to a research question using semantic similarity and provision of a summary (i) for each paper and (ii) based on the more relevant papers. Automation of the tasks of extracting data and synthesizing findings | Identification of relevant papers and provision of a summary (i) for each paper and (ii) based on the more relevant papers. Provision of specific information from each individual paper by interaction with a chatbot | Provision of quantitative and qualitative insight on how publications cite each other and how they refer to a specific research question. Identification of relevant papers and provision of a summary for each paper. |
| Number of sources | > 125 million sources (Semantic Scholar corpus) | > 200 million sources  (Semantic Scholar, OpenAlex, Google Scholar corpora) | > 200 million sources  (journals registered with Crossref or Datacite) |
| Presentation of gray literature | Preprints; conference abstracts | Preprints; conference abstracts | Preprints; conference abstracts |
| Presentation of results not written in English | Yes | Yes | Yes |
| Features of the paid version that can improve the search process | Export of results; explanations for AI-generated answers | AI models of “higher quality”; export of results | No free version available |

**Supplementary Table 2. GRADE evidence profile for the comparison between mometasone and placebo considering the results obtained with the main strategy and not considering the publication bias domain**

| **Source** | **Certainty assessment** | | | | | | **№ of patients** | | **Effect** | **Certainty** |
| --- | --- | --- | --- | --- | --- | --- | --- | --- | --- | --- |
|  | **№ of studies** | **Study design** | **Risk of bias** | **Inconsistency** | **Indirectness** | **Imprecision** | **Mometasone** | **Placebo** | **Absolute (95% CI)** |  |
| **TNSS [perennial allergic rhinitis] (follow-up: 4 weeks; Scale from: 0 to 12)** | | | | | | | | | | |
| AI-based main strategy | 1 | randomised trials | not serious | not serious | very serious^a^ | very serious^b^ | 20 | 10 | MD 2.4 lower (4.8 lower to 0 higher) | ⨁◯◯◯ Very low |
| Systematic review^11^ | 3 | randomised trials | serious^c^ | serious^d^ | very serious^a^ | serious^e^ | 211 | 194 | MD 1.1 lower (2.7 lower to 0.5 higher) | ⨁◯◯◯ Very low |
| **TNSS [seasonal allergic rhinitis] (follow-up: 2 weeks; Scale from: 0 to 12)** | | | | | | | | | | |
| AI-based main strategy | 8 | randomised trials | not serious | not serious | not serious | not serious | 1280 | 1086 | MD 1 lower (1.2 lower to 0.8 lower) | ⨁⨁⨁⨁ High |
| Systematic review^11^ | 17 | randomised trials | not serious | serious^d^ | not serious | not serious | 4225 | 2642 | MD 1 lower (1.1 lower to 0.8 lower) | ⨁⨁⨁◯ Moderate |
| **TOSS [seasonal allergic rhinitis] (follow-up: 2 weeks; Scale from: 0 to 9)** | | | | | | | | | | |
| AI-based main strategy | 2 | randomised trials | serious^f^ | not serious | serious^g^ | not serious | 514 | 495 | MD 0.4 lower (0.6 lower to 0.1 lower) | ⨁⨁◯◯ Low |
| Systematic review^11^ | 4 | randomised trials | serious^h^ | not serious | serious | not serious | 1078 | 918 | MD 0.3 lower (0.5 lower to 0.1 lower) | ⨁⨁◯◯ Low |
| **RQLQ [seasonal allergic rhinitis] (follow-up: 2 weeks; Scale from: 0 to 6)** | | | | | | | | | | |
| AI-based main strategy | 3 | randomised trials | serious^f^ | serious^d^ | serious^g^ | very serious^b^ | 622 | 611 | MD 0.5 lower (0.8 lower to 0.1 lower) | ⨁◯◯◯ Very low |
| Systematic review^11^ | 7 | randomised trials | serious^f^ | serious^d^ | serious^g^ | not serious | 1382 | 1226 | MD 0.4 lower (0.6 lower to 0.2 lower) | ⨁◯◯◯ Very low |

**CI:** confidence interval; **MD:** mean difference; **RQLQ:** Rhinoconjunctivitis Quality of Life Questionnaire; **TNSS**: Total Nasal Symptom Score; **TOSS**: Total Ocular Symptom Score; a. For all studies, spread measures had to be estimated. b. Small number of participants assessed and/or confidence interval crossing several decision thresholds; c. High risk of bias for the study with the highest precision; d. Substantial unexplained heterogeneity found; e. Small number of participants assessed and confidence interval crossing the decision threshold; ^f^ All studies displayed a high risk of bias; g. For several studies, spread measures and/or effect size measures had to be estimated; h. Only one study did not present a high risk of bias and in that study no significant difference was observed.

**Supplementary Table 3. Meta-analytical results obtained with different search strategies**

| **Platform** | **Study designs being queried** | **Queries with outcome indication** | **Meta-analytical point estimate (95% Confidence Interval) [*I*^2^] [*N* primary studies]** | | | |
| --- | --- | --- | --- | --- | --- | --- |
|  |  |  | **TNSS (SAR)** | **TOSS (SAR)** | **RQLQ (SAR)** | **TNSS (PAR)** |
| Multiple ^a^ | RCTs + SR | No | -0.98 (-1.22;-0.75) [43%] [8] | -0.36 (-0.58;-0.14) [0%] [2] | -0.47 (-0.96;0.02) [87%] [2] | -2.4 (-4.8;0) [0%] [1] |
| Multiple ^a^ | RCTs + SR | Yes | -0.98 (-1.22;-0.75) [43%] [8] | -0.36 (-0.58;-0.14) [0%] [2] | -0.46 (-0.77;-0.14) [71%] [3] | -2.4 (-4.8;0) [0%] [1] |
| Multiple ^a^ | RCTs | No + Yes ^b^ | -0.91 (-1.09;-0.72) [9%] [7] | -0.36 (-0.58;-0.14) [0%] [2] | -0.46 (-0.77;-0.14) [71%] [3] | -2.4 (-4.8;0) [0%] [1] |
| Multiple ^a^ | RCTs | No | -0.84 (-1.05;-0.63) [0%] [6] | -0.29 (-0.59;0) [0%] [1] | -0.23 (-0.45;-0.02) [0%] [1] | -2.4 (-4.8;0) [0%] [1] |
| Multiple ^a^ | RCTs | Yes | -0.86 (-1.11;-0.6) [23%] [4] | -0.36 (-0.58;-0.14) [0%] [2] | -0.46 (-0.77;-0.14) [71%] [3] | -2.4 (-4.8;0) [0%] [1] |
| Elicit | RCTs + SR | No + Yes ^b^ | -0.98 (-1.22;-0.75) [43%] [8] | -0.36 (-0.58;-0.14) [0%] [2] | -0.46 (-0.77;-0.14) [71%] [3] | -2.4 (-4.8;0) [0%] [1] |
| Elicit | RCTs + SR | No | -0.98 (-1.22;-0.75) [43%] [8] | -0.36 (-0.58;-0.14) [0%] [2] | -0.47 (-0.96;0.02) [87%] [2] | -2.4 (-4.8;0) [0%] [1] |
| Elicit | RCTs + SR | Yes | -0.98 (-1.22;-0.75) [43%] [8] | -0.36 (-0.58;-0.14) [0%] [2] | -0.46 (-0.77;-0.14) [71%] [3] | -2.4 (-4.8;0) [0%] [1] |
| Elicit | RCTs | No + Yes ^b^ | -0.86 (-1.11;-0.6) [23%] [4] | -0.36 (-0.58;-0.14) [0%] [2] | -0.46 (-0.77;-0.14) [71%] [3] | -2.4 (-4.8;0) [0%] [1] |
| Elicit | RCTs | No | -0.71 (-1.00;-0.41) [0%] [3] | -0.29 (-0.59;0) [0%] [1] | -0.23 (-0.45;-0.02) [0%] [1] | -2.4 (-4.8;0) [0%] [1] |
| Elicit | RCTs | Yes | -0.86 (-1.11;-0.6) [23%] [4] | -0.36 (-0.58;-0.14) [0%] [2] | -0.46 (-0.77;-0.14) [71%] [3] | -2.4 (-4.8;0) [0%] [1] |
| Scispace | RCTs + SR | No + Yes ^b^ | -1.01 (-1.22;-0.81) [0%] [5] | -0.44 (-0.77;-0.11) [0%] [1] | -0.73 (-1.01;-0.45) [0%] [1] | -2.4 (-4.8;0) [0%] [1] |
| Scispace | RCTs + SR | No | -1.01 (-1.22;-0.81) [0%] [5] | -0.44 (-0.77;-0.11) [0%] [1] | -0.73 (-1.01;-0.45) [0%] [1] | -2.4 (-4.8;0) [0%] [1] |
| Scispace | RCTs + SR | Yes | -1.01 (-1.22;-0.81) [0%] [5] | -0.44 (-0.77;-0.11) [0%] [1] | -0.73 (-1.01;-0.45) [0%] [1] | -2.4 (-4.8;0) [0%] [1] |
| Scispace | RCTs | No + Yes ^b^ | -0.97 (-1.26;-0.69) [0%] [4] | - [0] | - [0] | -2.4 (-4.8;0) [0%] [1] |
| Scispace | RCTs | No | -0.97 (-1.26;-0.69) [0%] [4] | - [0] | - [0] | -2.4 (-4.8;0) [0%] [1] |
| Scispace | RCTs | Yes | -0.92 (-1.68;-0.16) [0%] [1] | - [0] | - [0] | - [0] |
| Scite | RCTs + SR | No + Yes ^b^ | - [0] | - [0] | - [0] | - [0] |
| Scite | RCTs + SR | No | - [0] | - [0] | - [0] | - [0] |
| Scite | RCTs + SR | Yes | - [0] | - [0] | - [0] | - [0] |
| Scite | RCTs | No + Yes ^b^ | - [0] | - [0] | - [0] | - [0] |
| Scite | RCTs | No | - [0] | - [0] | - [0] | - [0] |
| Scite | RCTs | Yes | - [0] | - [0] | - [0] | - [0] |
| Results of the systematic review^7^ | | | -0.95 (-1.12;-0.79) [47%] [17] | -0.3 (-0.48;-0.12) [0%] [3] | -0.38 (-0.55;-0.2) [49%] [6] | -1.7 (-3.7;0.3) [59%] [3] |

PAR=Perennial allergic rhinitis; RCT=Randomised controlled trial; RQLQ=Rhinoconjunctivitis quality of life questionnaire; SAR=Seasonal allergic rhinitis; SR=Systematic review; TNSS=Total Nasal Symptom Score; TOSS=Total Ocular Symptom Score; ^a^ Results obtained when considering unique records from Elicit, Scispace and Scite altogether; ^b^ Results obtained when considering records obtained both with queries indicating and not indicating the outcomes of interest.

**Supplementary Table 4. Number of primary studies found with each strategy for searching AI-based platforms (i) when platforms were originally queried (“first search”), and (ii) when platforms were queried one month after (“second search”)**

| **Platform** | **Study designs being queried** | **Queries with outcome indication** | **First search: *N* eligible primary studies found** | **Second search: *N* eligible primary studies found** | ***N* eligible primary studies found in common** |
| --- | --- | --- | --- | --- | --- |
| **Main search strategy** | | | | | |
| All | RCTs + systematic reviews | Combination ^a^ | 18 ^a^ | 19 | 18 |
| **Additional search strategies** | | | | | |
| All | RCTs + systematic reviews | No | 17 | 18 | 17 |
| All | RCTs + systematic reviews | Yes | 17 | 19 | 17 |
| All | RCTs | Combination ^a^ | 17 | 18 | 17 |
| All | RCTs | No | 14 | 15 | 14 |
| All | RCTs | Yes | 12 | 13 | 12 |
| Elicit | RCTs + systematic reviews | Combination ^a^ | 18 | 18 | 18 |
| Elicit | RCTs + systematic reviews | No | 17 | 18 | 17 |
| Elicit | RCTs + systematic reviews | Yes | 17 | 18 | 17 |
| Elicit | RCTs | Combination ^a^ | 11 | 11 | 11 |
| Elicit | RCTs | No | 8 | 9 | 8 |
| Elicit | RCTs | Yes | 10 | 10 | 10 |
| Scispace | RCTs + systematic reviews | Combination ^a^ | 13 | 13 | 11 |
| Scispace | RCTs + systematic reviews | No | 12 | 13 | 10 |
| Scispace | RCTs + systematic reviews | Yes | 10 | 13 | 9 |
| Scispace | RCTs | Combination ^a^ | 11 | 11 | 11 |
| Scispace | RCTs | No | 10 | 10 | 10 |
| Scispace | RCTs | Yes | 4 | 3 | 3 |
| Scite | RCTs + systematic reviews | Combination ^a^ | 1 | 2 | 1 |
| Scite | RCTs + systematic reviews | No | 1 | 1 | 1 |
| Scite | RCTs + systematic reviews | Yes | 1 | 2 | 1 |
| Scite | RCTs | Combination ^a^ | 1 | 2 | 1 |
| Scite | RCTs | No | 1 | 1 | 1 |
| Scite | RCTs | Yes | 1 | 2 | 1 |

DOI=Digital object identifier; RCT=Randomised controlled trials; ^a^ Results obtained when considering records obtained both with queries indicating and not indicating the outcomes of interest

**Supplementary Table 5. Meta-analytical results obtained with the RCTs identified following the main strategy (i) when platforms were originally queried (“first search”), and (ii) when platforms were queried one month after (“second search”)**

| **Outcome** | **Meta-analytical results – first search: MD (95%CI) [*I*^2^] [*N* studies]** | **Meta-analytical results – second search: MD (95%CI) [*I*^2^] [*N* studies]** |
| --- | --- | --- |
| **TNSS (SAR)** | -0.98 (-1.22;-0.75) [43%] [8] | -0.98 (-1.18;-0.78) [38%] [9] |
| **TOSS (SAR)** | -0.36 (-0.58;-0.14) [0%] [2] | -0.30 (-0.48;-0.12) [0%] [3] |
| **RQLQ (SAR)** | -0.46 (-0.77;-0.14) [71%] [3] | -0.46 (-0.77;-0.14) [71%] [3] |
| **TNSS (PAR)** | -2.40 (-4.80;0) [-] [1] | -2.40 (-4.80;0) [-] [1] |

CI=Confidence interval; MD=Mean difference; PAR=Perennial allergic rhinitis; RQLQ=Rhinoconjunctivitis quality of life questionnaire; SAR=Seasonal allergic rhinitis; TNSS=Total Nasal Symptom Score; TOSS=Total Ocular Symptom Score

**Supplementary Table 6. Number of primary studies found with the main search strategy and with alternative search strategies for the case studies on olopatadine and olopatadine-mometasone**

| **Platform** | **Study designs being queried** | **Query with outcome indication** | **Olopatadine** | | **Olopatadine-mometasone** | |
| --- | --- | --- | --- | --- | --- | --- |
|  |  |  | ***N* unique records** | ***N* (%) of eligible primary studies found (*N*=6)** | ***N* unique records** | ***N* (%) of eligible primary studies found (*N*=4)** |
| **Main search strategy** | | | | | | |
| All | RCTs + systematic reviews | Combination ^a^ | 256 | 6 (100%) | 321 | 4 (100%) |
| **Additional search strategy** | | | | | | |
| All | RCTs + systematic reviews | No | 219 | 6 (100%) | 279 | 4 (100%) |
| All | RCTs + systematic reviews | Yes | 219 | 6 (100%) | 220 | 4 (100%) |
| All | RCTs | Combination ^a^ | 161 | 6 (100%) | 170 | 4 (100%) |
| All | RCTs | No | 123 | 6 (100%) | 122 | 4 (100%) |
| All | RCTs | Yes | 120 | 6 (100%) | 117 | 4 (100%) |
| Elicit | RCTs + systematic reviews | Combination ^a^ | 56 | 3 (50%) | 146 | 4 (100%) |
| Elicit | RCTs + systematic reviews | No | 50 | 3 (50%) | 134 | 4 (100%) |
| Elicit | RCTs + systematic reviews | Yes | 50 | 3 (50%) | 90 | 3 (75%) |
| Elicit | RCTs | Combination ^a^ | 56 | 3 (50%) | 63 | 3 (75%) |
| Elicit | RCTs | No | 50 | 3 (50%) | 50 | 3 (75%) |
| Elicit | RCTs | Yes | 50 | 3 (50%) | 50 | 3 (75%) |
| Scispace | RCTs + systematic reviews | Combination ^a^ | 158 | 6 (100%) | 150 | 4 (100%) |
| Scispace | RCTs + systematic reviews | No | 150 | 6 (100%) | 138 | 4 (100%) |
| Scispace | RCTs + systematic reviews | Yes | 154 | 6 (100%) | 127 | 4 (100%) |
| Scispace | RCTs | Combination ^a^ | 56 | 5 (83%) | 61 | 4 (100%) |
| Scispace | RCTs | No | 50 | 5 (83%) | 50 | 4 (100%) |
| Scispace | RCTs | Yes | 50 | 5 (83%) | 50 | 4 (100%) |
| Scite | RCTs + systematic reviews | Combination ^a^ | 77 | 2 (33%) | 106 | 3 (75%) |
| Scite | RCTs + systematic reviews | No | 50 | 2 (33%) | 73 | 3 (75%) |
| Scite | RCTs + systematic reviews | Yes | 50 | 2 (33%) | 74 | 3 (75%) |
| Scite | RCTs | Combination ^a^ | 77 | 2 (33%) | 83 | 2 (50%) |
| Scite | RCTs | No | 50 | 2 (33%) | 50 | 2 (50%) |
| Scite | RCTs | Yes | 50 | 2 (33%) | 50 | 2 (50%) |

RCT=Randomised controlled trials; ^a^ Results obtained when considering records obtained both with queries indicating and not indicating the outcomes of interest

**Supplementary Table 7. Number of primary studies found with the main search strategy and with alternative search strategies for the case study on the association between diabetes and tuberculosis**

| **Platform** | **Study designs being queried** | | ***N* unique records** | ***N* (%) of eligible primary studies found (N=48)** |
| --- | --- | --- | --- | --- |
| **Main search strategy ^a^** | |  | | |
| All | Cohort + systematic reviews | | 667 | 17 (35%) |
| **Additional search strategies ^a^** | |  | | |
| All | Cohort | | 134 | 4 (8%) |
| Elicit | Cohort + systematic reviews | | 404 | 15 (31%) |
| Elicit | Cohort | | 50 | 1 (2%) |
| Scispace | Cohort + systematic reviews | | 417 | 17 (35%) |
| Scispace | Cohort | | 50 | 3 (6%) |
| Scite | Cohort + systematic reviews | | 117 | 5 (10%) |
| Scite | Cohort | | 50 | 2 (4%) |

^a^ Considering the research question being addressed, all queries mentioned the outcome of interest

**Supplementary Table 8. Characteristics of the primary studies found obtained with the main search strategy for querying AI-based platforms *versus* the remaining studies included in the original systematic reviews assessing the association between diabetes and tuberculosis**

|  | **Identified studies (*N*=17)** | **Non-identified studies (*N*=31)** |
| --- | --- | --- |
| Publication year – median (minimum-maximum) | 2013 (2001-2020) | 2018 (2008-2023) |
| Absence of DOI – *N* (%) | 1 (5.9) | 3 (9.7) |
| Journal not indexed in MEDLINE – *N* (%)^a^ | 0 | 1 (3.2) |
| Absence of abstract – *N* (%) | 0 | 1 (3.2) |
| No explicit mention of the association between diabetes and tuberculosis in the title – *N* (%) | 6 (35.3) | 20 (64.5) |
| No explicit mention of the association between diabetes and tuberculosis in the title or abstract – *N* (%) | 2 (11.8) | 8 (25.8) |
| Outcome does not correspond to tuberculosis incidence – *N* (%) | 0 | 6 (19.4) |
| No explicit mention of the study design in title or abstract – *N* (%) | 3 (17.6) | 7 (22.6) |

^a^ MEDLINE searched via PubMed. All records were published in journals indexed in Google Scholar, Scopus and Web of Science.

**Supplementary Table 9. Main meta-analytical results for the association between diabetes and tuberculosis (i) in the original systematic review, and (ii) obtained with the main search strategy for querying AI-based platforms**

| **ES measure** | **Meta-analytical results – systematic review: ES (95%CI) [*I*^2^] [*N* studies]** | **Meta-analytical results – main search strategy: ES (95%CI) [*I*^2^] [*N* studies]** |
| --- | --- | --- |
| **HR** | 1.90 (1.51;2.40) [97%] [11] | 1.72 (1.41;2.09) [90%] [5] |
| **<10 years** | 1.52 (1.47;1.57) [0%] [7] | 1.65 (1.30;2.10) [54%] [2] |
| **≥10 years** | 2.44 (1.22;4.88) [99%] [4] | 1.76 (1.29;2.39) [95%] [3] |
| **OR** | 1.61 (1.27;2.04) [0%] [4] | - [-] [0] |
| **RR** | 1.60 (1.42;1.80) [0%] [8] | 1.75 (1.38;2.22) [0%] [5] |

CI=Confidence interval; ES=Effect size; HR=Hazard ratio; OR=Odds ratio; RR=Risk ratio

**Supplementary Table 10. Assessment of the query of the search strategy of the main case study using the checklist PRESS**

| **PRESS Checklist item** | **Completion (query of the main case study)** |
| --- | --- |
| Does the search strategy match the research question/PICO? | *Yes. The search strategy includes terms related to the population ([patients with] allergic rhinitis), intervention and study design.* |
| Are the search concepts clear? | *Search concepts are clear.* |
| Are there too many or too few PICO elements included? | *The search strategy includes terms related to the population ([patients with] allergic rhinitis), intervention and study design. The comparator (“placebo”) was not included due to being to generalist, and the outcomes were not included as that could lead to the exclusion of relevant studies.* |
| Are the search concepts too narrow or too broad? | *Search concepts appear adequate in terms of their broadness.* |
| Does the search retrieve too many or too few records? (Please show number of hits per line.) | *Number of hits per line not shown.* |
| Are unconventional or complex strategies explained? | *No unconventional or complex strategies applied. No explanation for the search strategy was provided.* |
| Are Boolean or proximity operators used correctly? | *Boolean or proximity operators used correctly. The only exception appears to be one line in the Embase query.* |
| Is the use of nesting with brackets appropriate and effective for the search? | *No inappropriate use of nesting with brackets identified.* |
| If NOT is used, is this likely to result in any unintended exclusions? | *NOT was just used to implement the search strategies for identifying randomized trials.* |
| Could precision be improved by using proximity operators (eg, adjacent, near, within) or phrase  searching instead of AND? | *Proximity operators are used for searches in MEDLINE, Embase and Web of Science.* |
| Is the width of proximity operators suitable (eg, might adj5 pick up more variants than adj2)? | *Given the context, the width of the proximity operators is relevant.* |
| Are the subject headings relevant? | *Subject headings are relevant.* |
| Are any relevant subject headings missing; for example, previous index terms? | *No relevant subject headings seem to be missing.* |
| Are any subject headings too broad or too narrow? | *Subject headings do not appear too narrow or too broad.* |
| Are subject headings exploded where necessary and vice versa? | *Except in the context of the validated search strategies, subject headings are always exploded.* |
| Are major headings (‘‘starring’’ or restrict to focus) used? If so, is there adequate justification? | *No major headings are applied.* |
| Are subheadings missing? | *Subheadings are not used except in validated search strategies. However, they do not seem to be missing.* |
| Are subheadings attached to subject headings? (Floating subheadings may be preferred.) | *No. Subheadings are not used.* |
| Are floating subheadings relevant and used appropriately? | *Not applicable.* |
| Are both subject headings and terms in free text (see the following) used for each concept? | *Yes. Subject headings are never used isolated for each concept.* |
| Does the search include all spelling variants in free text (eg, UK vs. US spelling)? | *Spelling variants in free text were included – e.g., “hayfever” vs “hay fever”; “pollinosis” vs “pollinosis”. In web of science, the UK variant for “randomization” (and related words) was introduced but not the US variant.* |
| Does the search include all synonyms or antonyms (eg, opposites)? | *Antonyms are not included. However, there do not seem to be relevant antonyms for the research question being addressed.* |
| Does the search capture relevant truncation (ie, is truncation at the correct place)? | *Truncation was set at the correct place.* |
| Is the truncation too broad or too narrow? | *No situations were identified in which truncation would be too broad or too narrow.* |
| Are acronyms or abbreviations used appropriately? Do they capture irrelevant material? Are the  full terms also included? | *Full terms are always included. Abbreviations commonly used in clinical practice have been included (e.g., “azeflu” for “azelastine-fluticasone”). The only missing relevant abbreviation would be “AR”, as it would lead to inclusion of a large amount of irrelevant material.* |
| Are the keywords specific enough or too broad? Are too many or too few keywords used? Are stop  words used? | *Keywords seem adequate in their broadness and number. Stop words are not used.* |
| Have the appropriate fields been searched; for example, is the choice of the text word fields  (.tw.) or all fields (.af.) appropriate? Are there any other fields to be included or excluded  (database specific)? | *The appropriate fields have been searched.* |
| Should any long strings be broken into several shorter search statements? | *No* |
| Are there any spelling errors? | *The following spelling error was identified: no space between “or” and “ryaltris” in the Embase query.* |
| Are there any errors in system syntax; for example, the use of a truncation symbol from a  different search interface? | *No syntax errors were identified.* |
| Are there incorrect line combinations or orphan lines (ie, lines that are not referred to in the final summation that could indicate an error in an AND or OR statement)? | *No orphan lines were identified.* |
| Are all limits and filters used appropriately and are they relevant given the research question? | *The filters implemented are justified by the study design.* |
| Are all limits and filters used appropriately and are they relevant for the database? | *The filters implemented are justified by the study design and relevant for the database.* |
| Are any potentially helpful limits or filters missing? Are the limits or filters too broad or too  narrow? Can any limits or filters be added or taken away? | *No potentially helpful limits or filters missing and the ones implemented (e.g., Cochrane Highly Sensitive Search Strategy for identifying randomized trials) is justified by the study design.* |
| Are sources cited for the filters used? | *The Cochrane Highly Sensitive Search Strategy for identifying randomized trials was used but not cited. The same was observed for other existing strategies for RCTs implemented in other bibliographic databases.* |

**Supplementary Box 1. Explanations and examples for each of the items of the checklist, with reporting items for authors using artificial intelligence (AI)-based platforms for evidence search**

**Item 1. Identify and justify the rationale and purpose for which AI-based platforms were used for evidence search (validation, complement or replacement of search in more traditional sources).**

**Explanation:** Searching on AI-based platforms can have multiple purposes. Firstly, such searches can help authors validate the queries that they intend to use in electronic bibliographic databases. Specifically, authors can test whether the queries that they have developed for searching electronic bibliographic databases identify all eligible records that would be identified by prompting AI-based platforms. Secondly, searching on AI-based platforms can complement more traditional search methods, such as searching electronic bibliographic databases. Finally, in time- and/or human- resource-constrained scenarios, searching AI-based platforms can be used to replace searches in “more traditional” sources.

**Example of adequate reporting:** “We conducted a systematic search (Table S1) on MEDLINE, Embase, Web of Science, and the Cochrane Central Register of Controlled Trials. We also manually searched the clinicaltrials.gov and the medRxiv platforms. As a complement to searches in those sources, we searched artificial intelligence-based platforms specialized on the identification of scientific publications.”

**Item 2. Specify all AI-based platforms queried – and, if available, their underlying models – and search dates.**

**Explanation:** AI-based search platforms have different algorithms and the latter are constantly evolving. Therefore, for transparency purposes, authors should specify the names of the AI-based platforms queried and the dates when searches were performed. In case platforms offer a free or paid version, that should also be reported alongside other potentially relevant options or features.

**Example of adequate reporting:** “We queried the free versions of two platforms that use AI to identify scientific articles: Elicit (elicit.com) and Scispace (typeset.io) (information on the underlying models not available) in November 24, 2024.”

**Item 3. Provide all the prompts used to query AI-based platforms.**

**Explanation:** The results provided by AI-based platforms vary according to the prompts that are used to query them. Therefore, in the same way that authors should make available the queries used for searching electronic bibliographic databases, the prompts for querying AI-based platforms should also be provided either in the main manuscript or as supplementary material. If additional filters or functionalities of the platforms are used (in addition to the prompts), authors should report them as well.

**Example of adequate reporting:** “We used the following prompt: ‘Indicate parallel randomized controlled trials comparing mometasone versus placebo on nasal symptoms, in adult patients with allergic rhinitis’”

**Item 4. Indicate whether there was a search for relevant systematic reviews and a subsequent retrieving of their references.**

**Explanation:** Identifying relevant systematic reviews and performing backward and forward citation tracking is a recommended approach when performing searches in the context of evidence synthesis studies. AI-based platforms can support the identification of systematic reviews addressing the question of interest. In our methodological framework, we point to the differences in the methodological steps to be followed to identify systematic reviews vis-à-vis primary studies of the design of interest.

**Example of adequate reporting:** “We also queried the aforementioned AI-based platforms to identify relevant systematic reviews (prompts in Supplementary Table 2). Two researchers independently screened (by title and – if needed – abstract) the first 50 results displayed by each platform and retrieved those corresponding to systematic reviews answering to the respective research question. We then performed backward and forward citation tracking by identifying all unique references and citations of the retrieved systematic reviews.”

**Item 5. Indicate and justify what was the number of records retrieved or screened from querying each platform.**

**Explanation:** Setting the number of records to evaluate when prompting AI-based platforms can be challenging. Depending on the context, authors may opt to either define (i) whether they will retrieve a predefined number of records or (ii) will be retrieving records until ceasing to identify relevant results. Nevertheless, to ensure transparency, authors should make this decision before platforms are queried and provide a supporting rationale.

**Example of adequate reporting:** “Considering that we would be expecting a large number of eligible references, we opted not to set a predefined number of records to evaluate when querying AI-based platforms. Instead, we kept on assessing retrieved records until ceasing to identify relevant results (we set as stopping rule the identification of 20 consecutive non-relevant records).”

**Item 6. Describe the results of the search process, providing the number of obtained records, the number of unique records and the number of relevant eligible studies.**

**Explanation:** As with other approaches for evidence search, authors should transparently document the results of their search process. A flow diagram can be used to present such results.

**Example of adequate reporting:** “By searching specialized AI-based platforms, we retrieved 200 records, of which 127 were unique. Among those, we identified five eligible studies, of which one had not been identified using other search approaches.”

**Supplementary Figure 1. Number of identified eligible primary studies according to the order of results returned by artificial intelligence (AI)-based platforms.** Each line represents a different query in an AI-based platform.
